# The multifaceted impact of structured training program on persons with Parkinson disease and their adult caregiver: A protocol for a systematic review

**DOI:** 10.1097/MD.0000000000033966

**Published:** 2023-07-14

**Authors:** Sharmila Gopala Krishna Pillai, Nor Azlin Mohd Nordin, Norlinah Mohamed Ibrahim

**Affiliations:** a Physiotherapy Program, Center for Rehabilitation and Special Needs Studies, Faculty of Health Sciences, Universiti Kebangsaan Malaysia, Kuala Lumpur, Malaysia; b Centre for Physiotherapy Studies, Faculty of Health Sciences, Universiti Teknologi MARA, Cawangan Selangor, Kampus Puncak Alam, Puncak Alam, Selangor, Malaysia; c Department of Medicine, Faculty of Medicine, Universiti Kebangsaan Malaysia, Kuala Lumpur, Malaysia.

**Keywords:** caregiver, knowledge, mobility, quality of life, structured training

## Abstract

Training caregivers and persons with Parkinson Disease (PwPD) is crucial to provide them with adequate knowledge and care skills in preparation for the Parkinson disease (PD) progression. This review will systematically evaluate the effect of structured training programs on the mobility and quality of life (QoL) of PwPD. In addition, the effect of such training programs on the QoL, burden of caregiving, and knowledge of PD among adult caregivers of PwPD will also be evaluated.

**Methods:**

Systematic and comprehensive search of relevant studies will be conducted using electronic databases such as Cochrane Library, EBSCOhost, PubMed, SCOPUS, and Web of Science. The title, abstract, keywords, and full texts will be screened for eligibility. Studies to be selected are randomized controlled trials (RCT) from inception until April 2023. Studies based on structured PD training either in the form of training, education, program, multidisciplinary approach, or self-management targeted at both PwPD and their adult caregivers will be selected. Only full-text articles available in the English language will be included. Full-text articles will be inspected by 2 independent reviewers to produce the final set of articles that meet the eligibility criteria. A third reviewer will be engaged if no consensus is achieved between the first and second reviewers. Version 2 of the Cochrane risk-of-bias tool for randomized trials (RoB 2) will be used to evaluate the quality of papers and inform the risk of bias.

**Results:**

This review will provide an outlook on the effects of structured PD training programs on mobility and QoL of PwPD. In addition, it will provide insight into the effects of such training on the caregivers’ burden, knowledge of PD, and QoL.

**Conclusion:**

This review findings may help clinicians and researchers to understand the effect of structured and comprehensive PD training programs for PwPD and their adult caregiver.

## 1. Introduction

Parkinson disease (PD) is a progressive neurodegenerative disorder characterized by motor and non-motor features. The disease progression is contributed by progressive neurodegeneration of numerous regions in the brain and peripheral nervous system.^[1,2]^ PD imposes mobility limitation which causes devaluation, sadness, and frustration among persons with Parkinson Disease (PwPD).^[3]^ Zhao et al^[4]^ reported PwPD demonstrated a lower quality of life (QoL) than healthy controls, particularly in physical function and mental health. A similar trend is observed in Malaysia with the non-motor aspect of PD greatly contributing to the decline in QoL in PwPD as measured by Parkinson Disease Questionnaire-39. The caregivers’ burden was also influenced by Parkinson Disease Questionnaire-39 QoL dimensions such as “mobility,” “activities of daily life,” “emotional well-being,” and “stigma” of PwPD.^[5]^

The progressive nature of PD with increasing disabilities imposes a significant burden on caregivers especially family members, being the main caregiver. Literature documented high occurrences of depression and anxiety among the caregivers of PwPD, which corresponds to the caregiving burden.^[6]^ Due to the chronic and progressive nature of PD with accompanying disabilities which worsen over time, care for this population is a significantly challenging task.^[7]^ The caregivers often experience long-term emotional and physical burden across all stages which eventually threatens their well-being and QoL. These, in return, may reduce the quality of care provided to PwPD and consequently, affect the functional level and general health of PwPD.^[8]^ Therefore, caregivers’ well-being deserves attention to minimizing these consequences. To address these concerns, training involving caregivers of PwPD is important to equip them with adequate knowledge and care skills particularly as the disease progresses.^[9]^

Training of PwPD and their caregivers is vaguely described in works of literature as it ranges from self-management program, educational program, and even multidisciplinary rehabilitation program. Self-management programs have reported the inclusion of training components.^[10]^ A systematic review^[11]^ emphasized the importance of incorporating training in an educational program to prompt and sustain behavioral changes. Recent literature^[12]^ also reports the inclusion of training from physiotherapy, speech therapy, and dance as part of the multidisciplinary program for PwPD. Hence, the operational definition of training in the rehabilitation of PD needs to be consolidated in the literature for the homogeneity of its description. Despite the availability of literature emphasizing training either in the form of self-management, educational programs, and even multidisciplinary, the distinct feature of structured training is subject to argument.^[11]^

A few systematic reviews either evaluating the effect of self-management programs,^[10,11,13]^ multidisciplinary rehabilitation programs,^[14,15]^ or integrative care^[16]^ on PwPD have been documented. The review by Tennigkeit et al^[11]^ is the only review to date evaluating the effect of a structured self-management program on PwPD. The review summarized details of interventions appearing structured with the use of professional teaching manuals, teaching manuals and handouts, and “homework or home exercise programs in between single modules.” In the review, 3^[17–19]^ studies with professional teaching manuals, 1^[20]^ study using teaching manuals and handouts, and 1^[21]^ using “homework or home exercise programs in between single modules” included caregivers. Overall, the review reported 35% of the programs in the review included caregivers.^[11]^ This highlights despite an attempt to review a structured self-management program consisting of training, this review did not solely focus on papers involving both PwPD and their caregivers.

Similarly, another review^[13]^ on self-management support programs for PwPD stated 4 interventions^[20,22–24]^ reported the inclusion of caregivers. Limited studies involving caregivers may be insufficient to conclude the benefit of such training mainly owing to its heterogeneous characteristics. One study reported the inclusion of persons with other neurological conditions^[20]^ while other studies exhibit key components of the program either solely education,^[22]^ education and problem-solving^[24]^ or education, problem-solving, and goal setting^[20,23]^ without the specificity of training in these articles. Outcomes varied in the review^[13]^ with the most common outcomes used were QoL, mental health status, and functions while only 1 study^[24]^ reported on PD knowledge. Another similar systematic review^[10]^ focuses also on self-management with common outcome measures such as QoL, well-being, and functional outcome measures, with only 7 of 36 studies included in the review involved caregivers. Despite suggesting a trend to support the inclusion of caregivers in the self-management program, the evidence is inconclusive^[10]^ warranting further study reviewing the involvement of the caregivers in the management of PwPD through structured training on various measures not limited to QoL.

A review^[14]^ reporting a multidisciplinary care model for PwPD in recent years concludes multidisciplinary care model may yield improvement in health-related QoL (HRQoL) and Unified Parkinson Disease Rating Scale while another review^[15]^ shows no superiority between the multidisciplinary and conventional care model on disability, functionality, and QoL despite improvement in carer anxiety level following multidisciplinary care. However, the review of Li et al^[14]^ only included 1 published article explicitly stating training in their intervention description with no inclusion of caregivers. Further, only 2 studies^[25,26]^ in the review reported caregiver strain. The review of Seid et al^[15]^ utilized Physiotherapy Evidence Database scale for methodological quality assessment of the reviewed papers which does not report the risk of bias of papers as suggested by Preferred Reporting Items for Systematic Reviews and Meta-Analyses guidelines which challenge the strength of the evidence. Another review^[16]^ reporting on integrative care provided for PwPD included only 2 articles and describe the effectiveness of mutidisciplinary care based on the studies. Only 2 studies however, may be insufficient to provide conclusion on the framework of integrative care and its effect.

There is no review of structured training programs involving both PwPD and their caregivers. Common outcome measures in the review were limited to QoL, function, and well-being, with minimal studies focusing on caregiver strain. The effect of such training on outcomes such as mobility, knowledge, and caregiver burden is still limited and lacking. The gaps arising following the comparison of reviews warrants the need for a review that comprehensively evaluates structured training programs for PwPD and their caregiver on various measures such as mobility, QoL, caregiver burden, and PD knowledge. Therefore, this systematic review aims to evaluate the effect of structured training programs on the mobility and QoL of PwPD plus the effect of such training on QoL, caregiving burden, and knowledge of PD among their caregivers.

## 2. Materials and methods

### 2.1. Study protocol registration

This systematic review protocol is based on the checklists of Preferred Reporting Items for Systematic Reviews and Meta-Analysis Protocol. This review is registered in the International Prospective Register of Systematic Reviews with registration number CRD42023394050 on March 16th, 2023.

### 2.2. Inclusion and exclusion criteria

The inclusion criteria of the articles to be included in this review are articles with randomized controlled trial (RCT) study design, which enrolled PwPD aged 18 years old and above & their adult caregiver, published as full-text in the English language, with structured PD training program, evaluating mobility and/or QoL of PwPD, which measured caregiver burden and/or QoL and/or PD knowledge. Structured PD training accepted in this review will be either in the form of training, education, program, multidisciplinary approach, or self-management targeted at both PwPD and their informal or formal caregivers. The term “structured” in structured PD training is described as either formal/standardized/detailed training following a clearly outlined structure/goal/theme in PD management. To include a variety of articles, articles evaluating functions specific to mobility will also be included. RCT study design is inclusive of both true and quasi-RCT.

This review will, however, exclude articles that solely evaluate the effect of an intervention and include participants of mixed neurological disorders besides PD. Intervention in this review is defined as treatment in the form of exercise, therapy, or rehabilitation without meeting the criteria of training as described which is either training, education, program, multidisciplinary approach, or self-management programs.

### 2.3. Search strategy and information sources

Articles search will be conducted using electronic databases such as Cochrane Library, EBSCOhost, PubMed, Scopus, and Web of Science using the following key search terms: “Parkinson,” “caregiver,” and “structured training.” More detailed search strategies are provided in Table 1. Articles will be searched and retrieved from the mentioned databases from the inception until April 2023. The reference list of identified records will then be screened for duplicates.

**Table 1:** Search strategy for Cochrane library, EBSCOhost, PubMed, SCOPUS, Web of Science.

No	Search strategy
#1	(Parkinson*)
#2	(Caregivers OR carer OR family)
#3	(structured training OR education OR program OR multidisciplinary OR self-manage*)
Combination search	#1 AND #2 AND #3
Limiters	Humans, English, Article, Full Text

### 2.4. Process of study selection

**Figure 1.**
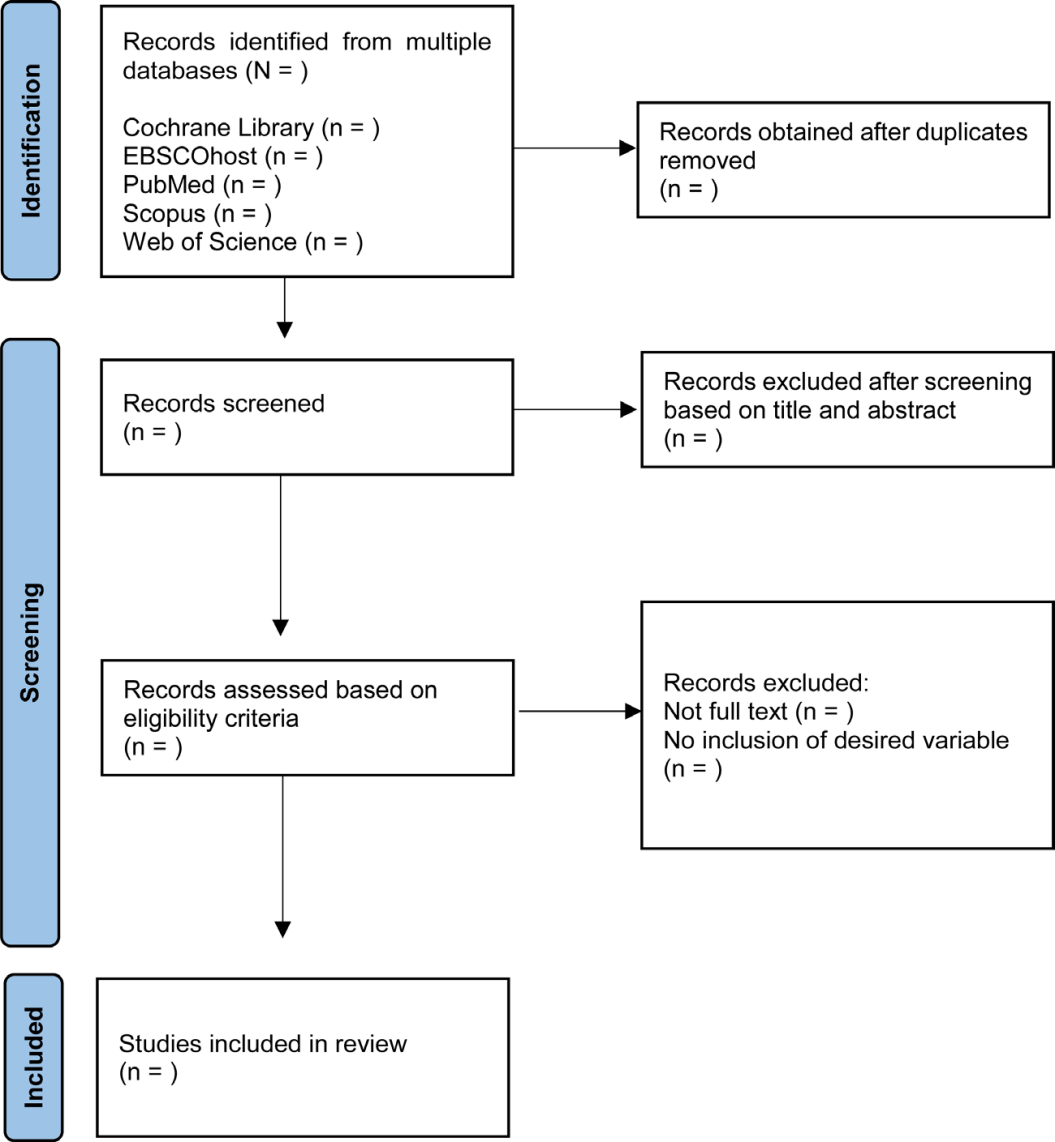
PRISMA flow chart of the study selection process in this review. PRISMA = Preferred Reporting Items for Systematic Reviews and Meta-Analyses.

The Systematic Review Data Repository will be used for data management in this review as it is a relational database promoting the development of data extraction forms for structured data collection consisting of an assessment that involves the risk of bias.^[27]^ Systematic review data repository is not only developed for data extraction, organization, and tabulation but for its distinctive archival feature which facilitates global collaboration and useful during the systematic review update.^[27]^ The selection of studies involves developing search strings for databases search. Obtained search results will be screened to remove duplicates. Initial screening will be conducted based on the title followed by the abstract. Once full-text articles are obtained and scrutinized by the 2 independent reviewers, the final set of articles that meets the criteria will be generated. However, a third reviewer will be engaged if no consensus is achieved. Preferred Reporting Items for Systematic Reviews and Meta-Analyses flow chart for systematic review and meta-analysis will be adhered in the study selection process (see Fig. 1).

### 2.5. Data extraction

One independent reviewer will extract data using a predetermined data collection form. The corresponding author of any study with missing or incomplete data will be contacted for further information. The data extraction form will record information regarding the following; study details consisting of information on the authors, date of the published study, location of study and setting of the training, study design and stratification if present, participants’ information including sample size, age and gender of participants in the study for both PwPD and their caregiver. For PwPD, information on the characteristics, type, and stage of PD will be recorded, clinical outcome measures, training information such as content and its parameters, feasibility and safety issues of such training.

### 2.6. Risk of bias

The instrument to evaluate the risk of bias in RCT will be Version 2 of the Cochrane risk-of-bias tool for randomized trials (RoB 2). RoB 2 consists of 5 bias domains namely “bias arising from the randomization process”, “bias due to deviations from intended interventions,” “bias due to missing outcome data,” “bias in the measurement of the outcome,” “bias in the selection of the reported result.” The assessment of each domain comprises a sequence of signaling questions, judgement on the bias risks assisted by an algorithm that connects the responses to signaling questions to a proposed judgement, and free text boxes to justify this mapping. There are also free text boxes to explain the direction of bias which is optional.^[28]^ RoB 2 will also be used to evaluate the risk of bias of “Quasi randomized trial” (closely mimicking RCT) as per Cochrane Handbook.^[29]^ One reviewer will use the appropriate tool to independently rate each included study. If queries or discrepancies regarding data extraction occur, these will be resolved by discussion with another reviewer. If not resolved, an independent researcher will complete the risk of bias tool, and a discussion will be made between the first and second authors to achieve consensus and to make the final decision.

### 2.7. Data synthesis

This review will report data on the changes in the score of mobility and QoL of PwPD plus the changes in the score of caregivers’ burden, QoL, and knowledge of PD from baseline to the last available follow-up. Data that will be synthesized from the retrieved papers will include study details (authors, date, location, setting, study design), participants’ information including sample size, age and gender of PwPD plus the characteristics of PD, sample size, age and gender of adult caregivers plus their relationship with PwPD, clinical outcome measures used (name of outcome measurement tools used), training parameters (frequency, intensity, repetition, volume, any relevant information for training dosage), training content (type, description, approach and structure of structured PD training), feasibility and safety issues (adverse events and the number of dropouts reported in total number), results for clinical measures (means, standard deviations, *P* values, effect size, medians and interquartile ranges, where appropriate).

### 2.8. Ethics

This review does not require ethical approval as only data from published studies will be used.

## 3. Discussion

Statistics of people suffering from PD will continue to increase, as does the duration of the condition, resulting in greater PD sufferers in advanced stages.^[30]^ PwPD experiences great limitations in functional mobility, and according to Bouça-Machado et al,^[3]^ patients feel embarrassed in public settings and under-appreciated due to the changeable nature of their illness with the presence of tremors and functional restrictions. The functional decline, however, correlates with cognitive decline in PD, and the presence of motor disability in PD may have an impact on it.^[31]^ In a recent study by Lubomski et al^[32]^ PwPD exhibits a lower HRQoL, with negative effects on HRQoL from both motor symptoms and non-motor symptoms. Similarly, a study in Malaysia reported QoL of PD is lower than the non-PD control group^[33]^ with non-motor PD symptoms affecting QoL greatly.^[34]^ This indicates the relationship of limitation in functional mobility to the overall function and QoL of PwPD. To address the PwPD diverse demands, optimal PD therapy necessitates a multidisciplinary approach and the involvement of many healthcare providers.^[7]^ The such approach needs to target the early onset of PD as the duration of PD is deemed an important risk factor for dementia among PwPD.^[35]^ The longer duration of PD and its progressive nature which causes severity also correlate with hospital admission.^[36]^

Caregivers’ involvement in the management of PwPD is undeniable. However, they face an immense burden following caregiving.^[37]^ Caregiver burden is defined as the perceived effort and stress that comes with having to care for a loved 1 with PD.^[38]^ The burden of caregiving for PwPD is influenced by PwPD and caregivers’ characteristics. This encompasses the HRQoL of PwPD, their spiritual health, their carers’ anxiety and depression, and their views of the patient QoL.^[39]^ Factors that are connected to caregivers’ overall QoL and HRQoL, upset caregivers and lower their QoL. These elements, which can be categorized as sociodemographic, psychological, and disease-related, are connected to the characteristics of both patients and caregivers.^[40]^

To date, the systematic reviews in the area of structured PD training consist of a self-management program, a multidisciplinary program, and integrated care with training as one of its component. However, such reviews fail to address both caregivers and PwPD as most studies included only PwPD or with minimal studies involving caregivers for a conclusive findings. Structured training for PwPD also needs to be defined conceptually, as at present only 1 review evaluating the effect of structured self-management in PwPD is available. The scarcity of evidence warrants a review that evaluates structured training for PwPD and caregivers. Such review will also be able to provide the principles and structure of training for this population and their caregiver to ensure homogeneity in the execution of the training. This will enable healthcare practitioners to develop structured training for PwPD and their caregivers to be well-prepared for the disease and its progression.

## Author contributions

**Conceptualization:** Sharmila Gopala Krishna Pillai, Nor Azlin Mohd Nordin, Norlinah Mohamed Ibrahim.

**Data curation:** Sharmila Gopala Krishna Pillai.

**Formal analysis:** Sharmila Gopala Krishna Pillai.

**Funding acquisition:** Nor Azlin Mohd Nordin.

**Investigation:** Sharmila Gopala Krishna Pillai.

**Methodology:** Sharmila Gopala Krishna Pillai, Nor Azlin Mohd Nordin.

**Project administration:** Sharmila Gopala Krishna Pillai.

**Resources:** Sharmila Gopala Krishna Pillai.

**Software:** Sharmila Gopala Krishna Pillai.

**Supervision:** Nor Azlin Mohd Nordin, Norlinah Mohamed Ibrahim.

**Validation:** Nor Azlin Mohd Nordin.

**Visualization:** Sharmila Gopala Krishna Pillai, Nor Azlin Mohd Nordin.

**Writing – original draft:** Sharmila Gopala Krishna Pillai.

**Writing – review & editing:** Sharmila Gopala Krishna Pillai, Nor Azlin Mohd Nordin.
